# High recurrence rate supports need for secondary prophylaxis in non-HIV patients with disseminated *mycobacterium avium* complex infection: a multi-center observational study

**DOI:** 10.1186/s12879-016-1411-8

**Published:** 2016-02-10

**Authors:** Siddharth Sridhar, Kitty S. C. Fung, Jasper F. W. Chan, Jimmy Y. W. Lam, Eric K. T. Yip, Ivan F.N. Hung, Alan K. L. Wu, Tak-Lun Que, Susanna K. P. Lau, Patrick C. Y. Woo

**Affiliations:** 1 State Key Laboratory of Emerging Infectious Diseases, The University of Hong Kong, Hong Kong, China; 2 Department of Microbiology, The University of Hong Kong, Hong Kong, China; 3 Research Centre of Infection and Immunology, The University of Hong Kong, Hong Kong, China; 4 Carol Yu Centre for Infection, The University of Hong Kong, Hong Kong, China; 5 Department of Microbiology, United Christian Hospital, Hong Kong, China; 6 Pamela Youde Nethersole Eastern Hospital, Hong Kong, China; 7 Department of Microbiology, Tuen Mun Hospital, Hong Kong, China; 8 Division of Infectious Disease, Department of Medicine, The University of Hong Kong, Hong Kong, China

**Keywords:** *Mycobacterium avium* complex, Non-HIV immunosuppression, Disseminated infection recurrence rate, Primary prophylaxis, Secondary prophylaxis

## Abstract

**Background:**

Long-term outcomes in non-HIV immunocompromised patients with disseminated *Mycobacterium avium* complex (dMAC) infections are unknown and the need for post-treatment secondary prophylaxis against MAC is uncertain in this setting. The objective of this study was to determine the need of continuing secondary anti-MAC prophylaxis in non-HIV patients after completing treatment of the primary dMAC episode.

**Methods:**

We conducted a ten-year multi-center analysis of non-HIV immunosuppressed patients with dMAC infections in Hong Kong.

**Results:**

We observed sixteen patients with dMAC during the study period of which five (31 %) were non-HIV immunosuppressed patients. In the non-HIV immunosuppressed group, three patients completed a treatment course without secondary prophylaxis, one patient received azithromycin-based secondary prophylaxis and one patient was still receiving therapy for the first dMAC episode. All the three patients who completed treatment without being given secondary prophylaxis developed recurrent dMAC infection requiring retreatment.

**Conclusions:**

In view of the high rate of dMAC infection recurrence in non-HIV immunocompromised patients following treatment completion, our data support long-term anti-MAC suppression therapy after treatment of the first dMAC infection episode in immunocompromised non-HIV patients, as is recommended for patients with advanced HIV. Tests of cell mediated immune function need to be evaluated to guide prophylaxis discontinuation in non-HIV patients.

## Background

Disseminated *Mycobacterium avium* complex (dMAC) infection is indicative of profound cell-mediated immunodeficiency and is best described in the setting of advanced HIV disease. However, with an expanding population of immunosuppressed patients worldwide, dMAC infections in the non-HIV population are increasingly being recognized in the scientific literature. Organ transplantation, immunosuppressants, novel immunomodulatory agents, autoimmune disorders, autoantibodies against interferon (IFN)-γ and hereditary immunological defects have all been associated with dMAC infection in HIV-negative individuals [1–7]. As the incidence of dMAC infection in the HIV-infected population declines due to effective combined antiretroviral therapy (cART) and anti-MAC primary and secondary prophylaxis [8], infections in the non-HIV population are likely to constitute an increasing proportion of the overall dMAC disease burden.

In HIV-positive patients with dMAC disease who respond to anti-MAC therapy, current guidelines advise the continuation of anti-MAC treatment as post-treatment suppression (secondary prophylaxis) until sustained CD4^+^ T-lymphocyte recovery to >100 cells/μL is achieved using combined antiretroviral therapy [9]. The anti-mycobacterial treatment regimens for non-HIV patients with dMAC infection are extrapolated from clinical experience in the HIV setting. However, the need for secondary prophylaxis to prevent dMAC infection recurrence after treatment completion remains a largely unaddressed issue in these patients. This is partly because anti-MAC prophylaxis recommendations for HIV patients are based on CD4^+^ T-lymphocyte measurements [9, 10] and cannot be easily applied to non-HIV patients who often suffer from diverse functional cell-mediated immune defects that are not easily quantified in routine clinical practice. Furthermore, a systematic description of long-term outcomes in non-HIV dMAC infection case cohorts is lacking and the rate of recurrence is unknown.

We addressed this issue by analyzing the epidemiology of patients with dMAC infections over a ten-year period in a multi-center study. We evaluated the outcomes of non-HIV patients with dMAC infection to determine the need for post-treatment secondary prophylaxis in non-HIV patients, as recommended in the HIV setting.

## Methods

This study was approved by the Institutional Review Board of The University of Hong Kong/ Hospital Authority Hong Kong West Cluster. Permission for retrospective data retrieval from clinical databases and waiver for informed consent were approved by the Institutional Review Board. We analyzed the records of patients with dMAC infections presenting between 2005 and 2014 to four public hospitals in Hong Kong. All four hospitals offer acute internal medicine services and one is a university teaching hospital providing hematopoietic stem cell and solid organ transplantation services. Patients were diagnosed with dMAC infection based on compatible clinical features and isolation of MAC from blood, bone marrow and/ or other extrapulmonary sterile sites. Patients with suspected specimen contamination who were not offered anti-mycobacterial treatment (e.g. single positive early morning urine culture) were excluded from the analysis. The clinical records of patients who fulfilled the inclusion criteria were examined in detail for demographic features, risk factors for opportunistic infections, clinical features, anti-mycobacterial regimen, prescription of anti-MAC secondary prophylaxis and long-term outcomes.

## Results

Sixteen patients fulfilling the criteria for dMAC infection were identified over the ten-year period. Five of sixteen (31 %) patients with dMAC infection were HIV-negative immunosuppressed patients, while the other eleven patients had HIV-AIDS. The clinical features of the non-HIV immunosuppressed patients with dMAC infection are summarized in Table 1 and described in detail below.

**Table 1:** Clinical features and outcomes in non-HIV patients with disseminated MAC infections

	Age^a^/sex	Underlying conditions	Presenting signs/symptoms	Site of MAC isolation	Treatment regime and duration	Time to recurrence^b^	Site of MAC at recurrence	Outcome^c^
1	85/M	Monoclonal gammopathy of undetermined significance	Fever, pain over sternum	Sternal mass biopsy tissue, sputum	Clarithromycin, ethambutol and rifampicin for 11 months	10 months	Skin nodules, sputum	Restarted on clarithromycin, ethambutol and rifampicin; died during treatment due to suspected lymphoma
2	43/F	Systemic lupus erythematosus on azathioprine	Fever, per vaginal bleeding, cutaneous nodules over legs, groin lymphadenopathy	High vaginal swab, groin lymph node, skin biopsy	Clarithromycin, ethambutol and rifampicin for 21 months	3 years	Bone marrow, femoral bone, knee joint fluid	Repeat course of clarithromycin, ethambutol and rifampicin; still receiving treatment (as of March 2015)
3	67/F^d^	Autoantibody against IFN-γ, paraproteinemia	Fever	Blood, bone marrow	Azithromycin, ethambutol and rifampicin for 24 months Azithromycin secondary prophylaxis	No dMAC recurrence	N/A	Developed lymphoma and died 9 months after completion of anti-MAC treatment
4	42/F	Autoantibody against IFN-γ Sjogren’s syndrome, autoimmune myelofibrosis on prednisolone and mycophenolate mofetil	Fever, night sweats, pain over sternum	Sternal bone aspirate	Clarithromycin, ethambutol and rifampicin for 12 months	1 year	Blood, sputum	Repeat course of clarithromycin, ethambutol and rifampicin; still receiving treatment (as of March 2015)
5	43/F	Autoantibody against IFN-γ Systemic lupus erythematosus, mixed connective tissue disease	Fever, cough	Bone marrow, lung biopsy, sputum	Clarithromycin, ethambutol and rifampicin; still receiving treatment (as of March 2015)	N/A	N/A	Radiological improvement in consolidative changes of left lung on interval imaging

^a^age at diagnosis of dMAC infection

^b^time to recurrence refers to duration between completion of treatment of primary dMAC episode to symptom onset of the recurrent episode

^c^patients were followed up from time of diagnosis of primary dMAC episode till time of writing—March 2015

^d^adopted from reference

### Patient 1

An 85-year-old man, with history of ischemic heart disease and monoclonal gammopathy, presented with fever, cough and atypical chest pain. Chest radiography showed an ill-defined shadow in the upper paramediastinal region with cortical disruption of sternum. Computed tomography (CT) of the thorax confirmed a presternal mass along with a spiculated soft tissue mass in the posterior segment of the right upper lobe. Histology of the presternal mass showed a chronic inflammatory infiltrate and was positive for acid-fast bacilli (AFB) by Ziehl-Neelsen (ZN) stain. Biopsy tissue and sputum both yielded MAC. The patient was started on clarithromycin, ethambutol and rifampicin. He competed 11 months of anti-MAC treatment, responding well with sputum culture conversion and radiological improvement.

Ten months after cessation of therapy, he presented again with fever, weight loss, skin nodules and generalized lymphadenopathy. Sputum and skin biopsy yielded MAC. dMAC disease or an underlying lymphoma were considered to be the cause of the generalized lymphadenopathy, however the patient declined further invasive investigations due to debilitated condition and advanced age. He was restarted on clarithromycin, rifampicin and ethambutol, but eventually succumbed.

### Patient 2

A 43-year-old woman, with background history of systemic lupus erythematosus (SLE) on azathioprine, presented with pervaginal bleeding, enlarged left groin lymph node and indurated erythematous lesions over the lower limbs. Groin lymph node biopsy showed granulomatous inflammation with numerous AFB. The patient’s high vaginal swab and swabs from lower limb lesions both yielded MAC. HIV antibody was negative. She was started on rifampicin, clarithromycin and ethambutol.

Staging positron emission tomography—computed tomography (PET-CT) after fourteen months of anti-MAC treatment showed multiple soft tissue nodules of both lungs although histology and microbiology of specimens from these sites did not detect any AFB. In view of radiological suspicion of ongoing dMAC infection and immunosuppressed state, she received 21 months of anti-MAC treatment in total with end-of-treatment PET-CT documenting resolution of lung lesions and lymphadenopathy. She presented three years after cessation of treatment with recurrent dMAC infection involving the bone marrow, left distal femur and knee joint. She required repeated debridement and sequestrectomy of infected bone and knee joint and was treated with clarithromycin, rifabutin, ethambutol and moxifloxacin.

### Patient 3

A 67-year-old woman with good past health presented with pyrexia of unknown origin. PET-CT imaging showed multiple hypermetabolic active lesions over bilateral lung fields and generalized lymphadenopathy. Bone scintigraphy further showed multifocal active lesions in the vertebral column, bilateral ribs and right clavicle. Cultures of blood and bone marrow yielded MAC and she received a regimen containing azithromycin, rifampicin and ethambutol. She was confirmed to have autoantibody against IFN-γ.

Treatment was continued for two years and then switched to long-term secondary prophylaxis with azithromycin. She died due to lymphoma nine months after completing the multi-drug regimen for dMAC. Sputum, blood and urine cultures for acid-fast bacilli obtained before death were negative.

### Patient 4 (this patient was also described in reference 4)

A 42-year-old woman with background history of Sjogren’s syndrome and myelofibrosis, receiving mycophenolate mofetil and prednisolone 25 mg daily, presented with a sternal soft tissue mass. Fine needle aspiration cytology from the mass lesion was compatible with mycobacterial infection. Culture of the pus yielded MAC. PET-CT imaging showed hypermetabolic precarinal lymphadenopathy and extensive osseous hypermetabolic lesions involving multiple vertebrae, left scapula, sternum, right humeral head, bilateral ribs, ilia and pubic rami indicative of dMAC. Autoantibody against IFN-γ was positive.

She was started on a regimen comprising ethambutol, clarithromycin and rifampicin, completing one year of treatment with resolution of osseous lesions on reassessment imaging. One year after completing treatment, she presented with pyrexia of unknown origin. PET-CT showed a cavitatory lesion in upper lobe of left lung. Blood and sputum both yielded MAC; she was restarted on clarithromycin, rifampicin and ethambutol with good clinical response.

### Patient 5

A 43-year-old woman with SLE and mixed connective tissue disease presented with fever and cough for one month. CT of the thorax showed a left upper lobe mass lesion. Bronchoscopic biopsy of the lesion revealed a mixed active chronic inflammatory infiltrate with foci of granulomatous inflammation. AFB were demonstrated by ZN stain. Bone marrow examination was also performed in view of anemia (Hemoglobin: 6.8 g/dL) and leukocytosis (WBC: 36.29 × 10^9^/L) showing hypercellular marrow with mature plasma cells. Multiple sputum specimens, bronchial tissue and bone marrow yielded MAC confirming the diagnosis of dMAC.

As she was not on immunosuppressive treatment at presentation, immunological workup was performed confirming that she had autoantibody against IFN-γ.. She was started on clarithromycin, rifampicin and ethambutol; treatment of this patient is ongoing with good clinical and radiological response.

## Discussion

In this ten-year multi-center study that identified sixteen patients with dMAC, non-HIV patients accounted for approximately one-third (5/16) of all patients with dMAC infection over the study period, which constitutes a significant proportion of the overall dMAC disease burden in our setting. This finding is in sharp contrast to previous studies involving disseminated non-tuberculous mycobacterial disease cohorts conducted more than 15 years ago, which found that dMAC infections were almost entirely encountered in HIV-positive patients [11, 12]. With the fall in dMAC infection incidence in HIV carriers and the increasing population of non-HIV immunocompromised patients, it is expected that an increasing proportion of all dMAC infections will occur in non-HIV patients as was observed in our cohort.

This is the first systematic analysis of long-term outcomes in a non-HIV dMAC patient cohort. We found that all three non-HIV immunocompromised patients who were not prescribed secondary anti-MAC prophylaxis developed recurrent dMAC infection after treatment completion. Among the eleven HIV-positive dMAC patients, five completed treatment for the first dMAC episode while six died during the treatment period due to other opportunistic infections and malignancy. In contrast to the non-HIV patient group, none of the five HIV-positive patients who completed treatment for the first dMAC infection episode and received cART developed recurrence (data not shown). This finding is especially important as there is a lack of recommendations for secondary anti-MAC prophylaxis in the non-HIV immunocompromised population following treatment completion of the first episode of dMAC infection.

Based on the results of our study, we believe that patients with profound immunosuppression are likely to benefit from long-term secondary prophylaxis against MAC after treatment completion unless their immunological dysfunction is temporary and reversible. However, due to the heterogeneity of immunosuppressive conditions predisposing to dMAC infection in non-HIV patients, large clinical trials evaluating different prophylaxis strategies are likely to be infeasible. Therefore, we recommend that patients who have completed a course of anti-MAC therapy but deemed to have ongoing severe immunosuppression (e.g. high immunosuppressant dosages, primary immunodeficiencies, low CD4^+^ counts) should be continued on oral azithromycin and ethambutol as secondary prophylactic agents until such time as restoration of immunological function is achievable. This regimen of secondary prophylaxis has been shown to be safe and well tolerated in the HIV population. To reduce pill burden and improve patient compliance, azithromycin monotherapy for secondary prophylaxis may be considered for some patients who are deemed to be at ongoing risk for opportunistic infections but have improving immunological function (such as transplant recipients on tapering dose of immunosuppressants), but this strategy carries a risk of selecting out macrolide resistance in any residual MAC and, if used, should follow a duration of two-drug suppressive therapy. Breakthrough dMAC disease while on macrolide monotherapy prophylaxis should prompt clarithromycin susceptibility testing and an empirical dMAC treatment regimen that includes at least three antimycobacterial drugs active against MAC. Long term secondary prophylaxis may not be necessary in immunosuppressed patients with localized infection; we identified one patient who developed sternal osteomyelitis due to MAC without evidence of dissemination (unpublished data). She had no recurrence at ten years follow up despite not receiving secondary prophylaxis. Currently, there is insufficient evidence for primary anti-MAC prophylaxis in any non-HIV immunocompromised group, but this may be a consideration in patients with newly diagnosed anti-IFN-γ autoantibodies, a common risk factor for dMAC infection in our study.

In addition to quantitative counts of CD4^+^ T-lymphocytes, tests of T-lymphocyte function are required in routine clinical practice to objectively assess cell mediated immunological function of patients at treatment completion of dMAC to guide prophylaxis decisions. While classical lymphocyte proliferation assays are labor intensive and require specialist laboratory support, the clinical utility of newer tests of T-lymphocyte function such as the ImmuKnow assay (ViraCor-IBT, USA) and flow cytometry based proliferation assays are being explored [13, 14]. The role of monitoring markers of cell-mediated immunity to guide anti-MAC prophylaxis discontinuation in non-HIV populations deserves further evaluation.

## Conclusions

Recurrence of dMAC infections after treatment completion was very common in non-HIV immunosuppressed patients, warranting the consideration of post-treatment secondary prophylaxis as in their HIV-positive counterparts.

## Availability of data and materials

Not applicable.
